# Neoadjuvant Radiochemotherapy and Total Neoadjuvant Therapy in the Management of Locally Advanced Rectal Cancer

**DOI:** 10.7759/cureus.96517

**Published:** 2025-11-10

**Authors:** Mücahit Yigit, Siyer Roohani, Annika Kurreck, Robert Siegel, Dominik P Modest, David Kaul, Felix Ehret

**Affiliations:** 1 Radiation Oncology, Charité-Universitätsmedizin Berlin, Berlin, DEU; 2 BIH Biomedical Innovation Academy, BIH Charité (Junior) Clinician Scientist Program, Berlin Institute of Health (BIH) at Charité-Universitätsmedizin Berlin, Berlin, DEU; 3 Radiation Oncology, German Cancer Consortium (DKTK) Partner Site Berlin, a Partnership Between German Cancer Research Center (DKFZ) and Charité-Universitätsmedizin Berlin, Berlin, DEU; 4 Hematology, Oncology and Tumor Immunology, Charité-Universitätsmedizin Berlin, Berlin, DEU; 5 Surgery, Charité-Universitätsmedizin Berlin, Berlin, DEU; 6 Radiation Oncology, Health and Medical University, Potsdam, DEU

**Keywords:** locally advanced rectal cancer, neoadjuvant chemoradiotherapy, neoadjuvant radiochemotherapy, rectal cancer, total neoadjuvant therapy

## Abstract

Background: Neoadjuvant radiochemotherapy is a key treatment modality for locally advanced rectal cancer (LARC). Total neoadjuvant therapy (TNT) is a relatively new treatment approach consisting of neoadjuvant radiochemotherapy and consolidative or inductive chemotherapy followed by curative resection. In this study, we aimed to assess the treatment outcomes at a tertiary care center, focusing on neoadjuvant treatments, and to compare our results to the available literature.

Methods: This retrospective cohort study included patients with LARC receiving TNT or neoadjuvant radiochemotherapy without postoperative chemotherapy between 2014 and 2023.

Results: A total of 122 patients with LARC, with a median follow-up of 21.4 months, were included. Thirty-one patients received TNT (25.4%). Ninety-one patients (74.6%) received long-course radiochemotherapy. Sixty-eight patients (55.7%) were classified as high-risk. Twenty-six cases of distant metastases (21.3%) and nine cases of local recurrence (7.4%) were observed. Five-year progression-free and distant metastasis-free survival rates were 62.9% and 68.3%, respectively. The five-year local control was 92.7%. Poor post-treatment tumor regression grading (TRG) in the surgical specimen was found to be associated with worse progression-free survival (hazard ratio (HR): 2.55, p=0.028). Tumors in the lower rectum were associated with worse local control (HR: 5.70; p=0.044).

Conclusions: Neoadjuvant radiochemotherapy, including TNT, for the treatment of LARC appears to be a safe and effective treatment option. TRG may play a role in identifying patients at high risk of early progression. Further research is warranted to determine the optimal TNT sequence and to improve patient selection to avoid over- and undertreatment.

## Introduction

Rectal cancer is one of the most common solid malignancies and is responsible for a considerable proportion of cancer-related deaths [1]. Despite existing screening tools, such as the fecal occult blood test and colonoscopy, many tumors are diagnosed at a locally advanced stage [2]. Locally advanced rectal cancer (LARC) is commonly treated with neoadjuvant radiochemotherapy, surgical resection, and risk-adapted adjuvant chemotherapy, mostly utilizing folinic acid, fluorouracil (5-FU), and oxaliplatin (FOLFOX) or capecitabine and oxaliplatin (CAPOX) [2]. Recently, several neoadjuvant protocols shifted all nonsurgical treatment components into the neoadjuvant setting [2,3]. Total neoadjuvant therapy (TNT) can either consist of short-course preoperative radiotherapy (SC-TNT) with consolidation chemotherapy (e.g., nine cycles of FOLFOX) or long-course radiochemotherapy (LC-TNT) with either induction or consolidation chemotherapy (e.g., nine cycles of folinic acid, 5-FU, irinotecan, and oxaliplatin (FOLFIRINOX)) [2,3]. Similarly, conventional radiochemotherapy is a combination of long-course preoperative radiochemotherapy (LC-RCT) and concomitant 5-FU or capecitabine, often adding risk-adapted adjuvant chemotherapy (e.g., capecitabine monotherapy) [2,4]. In several prospective clinical studies, TNT has been found to have a notable progression-free survival (PFS) and distant metastasis-free survival (DMFS) benefit in comparison to LC-RCT and conventional adjuvant chemotherapy protocols [3,5]. Therefore, TNT became a focal point of interest for the treatment of LARC. The use of consolidative chemotherapy, in combination with radiotherapy, might lead to a reduction in micrometastases, thus reducing local recurrences and distant metastases [2,3]. Additionally, consolidative chemotherapy may affect radiation-resistant cells, which might have survived under conventional therapy protocols [1-3]. Regarding compliance, neoadjuvant chemotherapy has lower rates of complications and, subsequently, higher rates of completion [2]. Comparatively, conventional adjuvant chemotherapy is often less tolerated because of postoperative complications [2].

The widespread use of conventional LC-RCT was already well-established and incorporated in various guidelines. Comparatively, the PFS and DMFS superiority of TNT necessitated more detailed guidelines. The European Society for Medical Oncology (ESMO) recommends TNT for patients with the ESMO risk grouping "intermediate," "advanced," and "bad," a stratification based on TNM staging, extramural vascular invasion (EMVI), circumferential resection margin (CRM) positivity, as well as positive lateral lymph nodes [6]. The National Comprehensive Cancer Network (NCCN) recommends TNT for all patients with either cT3+ and/or cN1/2 [7]. While the ideal patient selection and the optimal TNT scheme are subjects of ongoing trials and research, we aimed to report our experience with the use of TNT and neoadjuvant treatments only for LARC at Charité-Universitätsmedizin Berlin, Germany, a tertiary care center, and compare our results with the available literature.

## Materials and methods

The inclusion criteria of this retrospective, single-center cohort study comprised: (i) patients with histopathologically confirmed LARC; (ii) neoadjuvant therapy consisting of either TNT or exclusively neoadjuvant conventional radiochemotherapy without adjuvant chemotherapy between 2014 and 2023; (iii) concluding surgical resection without adjuvant chemotherapy; and (iv) at least one available follow-up. Medical records were screened for the collection of patient, treatment, and tumor characteristics. Endpoints of interest were PFS, DMFS, overall survival (OS), and local control (LC). PFS was defined as the time period between the initial surgical resection and tumor progression, i.e., local recurrence, new metastasis, or death. Furthermore, DMFS was defined as the time between initial resection and the occurrence of any new distant metastasis or death. OS was the time between initial resection and death. LC was defined as the period between initial resection and local or locoregional recurrence or death of any cause. Follow-up data were collected from the institutional database or, if applicable, archived records, and stratified into clinical and radiographic follow-up. Patients without events were censored on the last day of imaging (radiographic follow-up) or clinical contact (clinical follow-up).

Previously reported prognostic factors for outcomes in LARC patients were assessed and utilized for endpoint analyses. These included pretreatment T4 stage, positive lymph nodes (either as cN positivity in ESMO or lateral positive lymph nodes in Rectal Cancer and Preoperative Induction Therapy Followed by Dedicated Operation (RAPIDO) trial), and a CRM ≤ 1 mm [3,8-11]. Notably, all the aforementioned variables, together with a positive EMVI, are classified as a high-risk category in the RAPIDO trial and as a clinical indication for TNT in the current ESMO guideline [3,6,10]. In our study, these variables were included in a singular high-risk category in line with the RAPIDO trial [3]. Given the varying findings regarding the role of EMVI in LC, we did not include EMVI status in the multivariable regression analysis for local tumor control [12]. Lower rectum location (located less than 6 cm from the anal verge) was described as a significant factor for decreased rate of LC but not for the occurrence of metachronous distant metastasis and worse OS [13]. Consequently, this factor was included in the analysis of LC and PFS. Tumor regression grading (TRG), according to Dworak et al. (1997), was assessed, and TRG response was stratified into two groups: TRG responders (grading 3-4) with a few to no malignant cells and TRG nonresponders (grading 1-2) with a significant amount of vital malignant cells in the post-treatment surgical specimen [14]. Time-to-event endpoints were analyzed using Kaplan-Meier estimates. Multivariable Cox regression analyses were performed, with an a priori selection of investigated variables as described above. The Cox proportional assumption was visually assessed using log-log plots and testing based on Schoenfeld residuals. P-values of ≤ 0.05 were considered statistically significant. Percentages, for instance, in descriptive statistics, might not add up due to rounding. Statistical analyses were done using IBM SPSS Statistics v27 (IBM Corp., Armonk, NY, USA) and GraphPad Prism v.9.5.0 (GraphPad Software, Inc., San Diego, CA, USA). The study was approved by the local institutional review board (EA1/236/23).

## Results

Patient and tumor characteristics

A total of 259 patients diagnosed with LARC who received surgical treatment during the study period were initially identified. After applying our inclusion criteria, 137 patients were excluded from the study. Specifically, 90 patients were excluded due to receiving adjuvant chemotherapy, 33 patients did not receive either neoadjuvant radiation or neoadjuvant chemotherapy, and 14 patients lacked either general treatment information or follow-up data. As such, 122 patients met the inclusion criteria and were retrieved for further analysis. Most patients were men (87 patients, 71.3%). The median age at first diagnosis was 63.5 years (range 25.4 to 82.8). Most tumors were located in the lower third of the rectum (48.4%). Most cases of LARC were adenocarcinoma (93.4%). The remaining were mucinous adenocarcinoma (4.1%), signet cell carcinoma (1.6%), and clear cell carcinoma (0.8%). The overwhelming majority of LARC cases were microsatellite stable (99.1%), with one exception of microsatellite instability (0.8%). More than half of the patients fulfilled the predefined high-risk criteria (55.7%) and were Union for International Cancer Control (UICC) stage III (86.1%). Positive lymph nodes (cN+) were detected in 98 patients (80.3%). Comparatively, positive lateral lymph nodes were present in 21 patients (17.2%).

**Figure 1:**
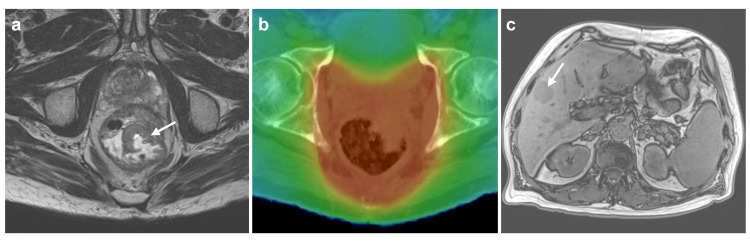
Case example Case example of a 69-year-old patient with UICC stage IIIC LARC, who underwent SC-TNT and R0 resection. (a) MRI showing the initial extension of the LARC (arrow). (b) Dose distribution of volumetric modulated arc therapy (axial image). (c) MRI demonstrating a singular liver metastasis (arrow), diagnosed approximately three months after surgery, after the patient complained about upper abdominal pain. UICC: Union for International Cancer Control; LARC: Locally advanced rectal cancer; SC: short-course; TNT: total neoadjuvant therapy

The neoadjuvant therapy model was as follows: 91 patients received conventional combined radiochemotherapy (74.6%), 27 patients received SC-TNT (22.1%), and four patients received LC-TNT (3.3%). The median prescription dose was 50.4 Gy (range 25 to 56 Gy) with a median fraction number of 28. Of the patients with consolidative or induction chemotherapy, most received a variation of the FOLFOX protocol (mostly FOLFOX4 or FOLFOX6; 77.4%). Patients undergoing conventional LC-RCT primarily received 5-FU or capecitabine monotherapy (94.7%). The median time between the start of chemotherapy and surgery was 3.5 months (interquartile range (IQR) 3.0 to 4.6). The surgical quality was high, with a median Mercury score of 1 (i.e., total mesorectal excision (TME) complete; 110 patients, 90.2%) and R0 resection status in most patients (118 patients, 96.7%). A pathological response, according to TRG, was achieved in a notable proportion of patients (49 patients, 40.2%), with pathological complete response (pCR) observed in 28.7% of all cases. Patient, tumor, and treatment characteristics are summarized in Table 1. A patient treatment case is illustrated in Figure 1.

**Table 1:** Patient, tumor, and treatment characteristics CRM: circumferential resection margin; EMVI: extramural vascular invasion; IQR: interquartile range; LC: long course (regarding radiation regimen); NA: not available; RCT: radiochemotherapy; SC: short course (regarding radiation regimen); TNT: total neoadjuvant therapy; UICC: Union for International Cancer Control; TRG: tumor regression grading; DPD: dihydropyrimidine dehydrogenase; 5-FU: fluorouracil; CAPOX: capecitabine and oxaliplatin; FOLFIRINOX: folinic acid, 5-FU, irinotecan, and oxaliplatin; FOLFOX: folinic acid, 5-FU, and oxaliplatin

Parameters	N	%
Median age (at time of initial diagnosis)	63.5 (range 25.4-82.8)	
Sex		
Male	87	71.3
Female	35	28.7
Primary tumor location		
Upper third	16	13.1
Middle third	47	38.5
Lower third	59	48.4
Tumor stage (UICC)		
II (unspecified)	3	2.5
IIA	13	10.7
IIB	1	0.8
III (unspecified)	29	23.8
IIIA	8	6.6
IIIB	52	42.6
IIIC	16	13.1
Histology		
Adenocarcinoma	114	93.4
Mucinous adenocarcinoma	5	4.1
Signet ring cell carcinoma	2	1.6
Clear cell adenocarcinoma	1	0.8
Tumor grading		
G1	3	2.5
G2	113	92.6
G3	6	4.9
DPD exon skipping mutation	1	0.8
High risk factors		
cT4	23	18.9
cN+	98	80.3
CRM (+)	45	36.9
EMVI (+)	15	12.3
Lateral (+) lymph nodes	21	17.2
Neoadjuvant therapy model		
SC-TNT	27	22.1
LC-TNT	4	3.3
LC-RCT	91	74.6
Delivered radiotherapy dose (in Gy)		
Median dose (IQR; range)	50.4 (45-50.4; range 18-56)	
Median fraction dose (IQR; range)	1.8 (1.8-2.0; range 1.8-5)	
Median fraction number (IQR; range)	28 (25-28; range 5-29)	
Discontinuation of radiation protocol	6	4.9
Concomitant chemotherapy	95	
5-FU	23	18.9
Capecitabine	67	54.9
CAPOX	2	1.6
Other/not specified	3	2.5
Induction/consolidation chemotherapy	31	
CAPOX	6	4.9
FOLFOX4	7	5.7
FOLFOX6	17	13.9
FOLFIRINOX	1	0.8
Dose reduction	12	38.7
Median number of cycles (IQR; range)	7 (6-9; range 1-12)	
Resection status		
R0	118	96.7
R1	4	3.3
Mercury score		
Median Mercury score	1	
Mercury 1	110	90.2
Mercury 2	6	4.9
Mercury 3	5	4.1
NA	1	0.8
TRG (Dworak)		
Median TRG	2	
TRG 4	35	28.7
TRG 3	14	11.5
TRG 2	44	36.1
TRG 1	27	22.1
TRG 0	2	1.6

Outcomes

**Figure 2:**
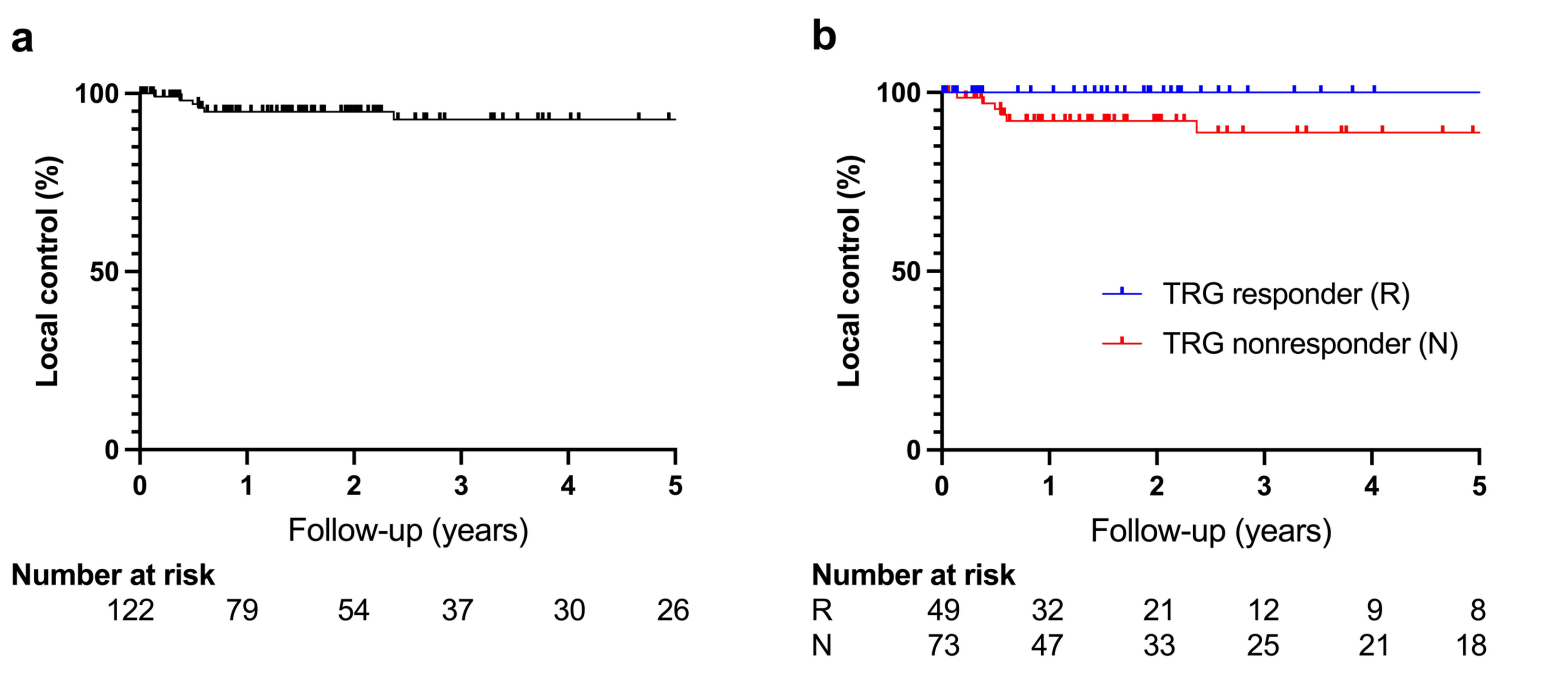
Local control (a) Local control for the whole study cohort. (b) Local control according to TRG response. TRG: tumor regression grading

**Figure 3:**
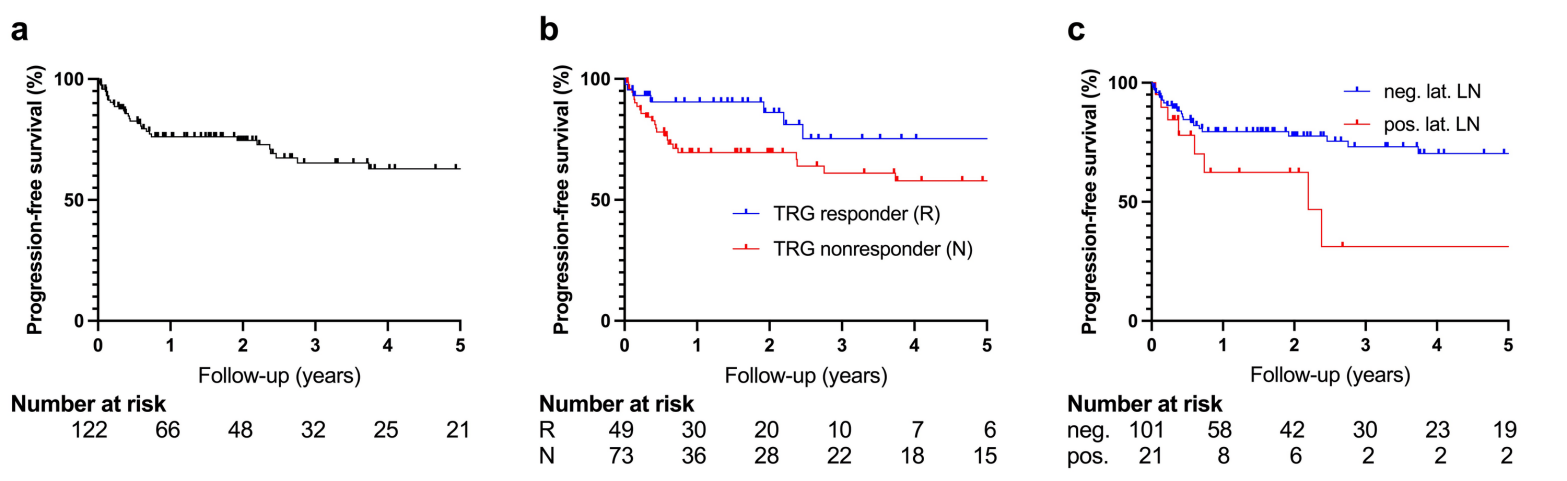
Progression-free survival (a) Progression-free survival for the whole study cohort. (b) Progression-free survival according to TRG response. (c) Progression-free survival according to lat. LN status. TRG: tumor regression grading; lat. LN: lateral lymph node; pos.: positive; neg.: negative

**Figure 4:**
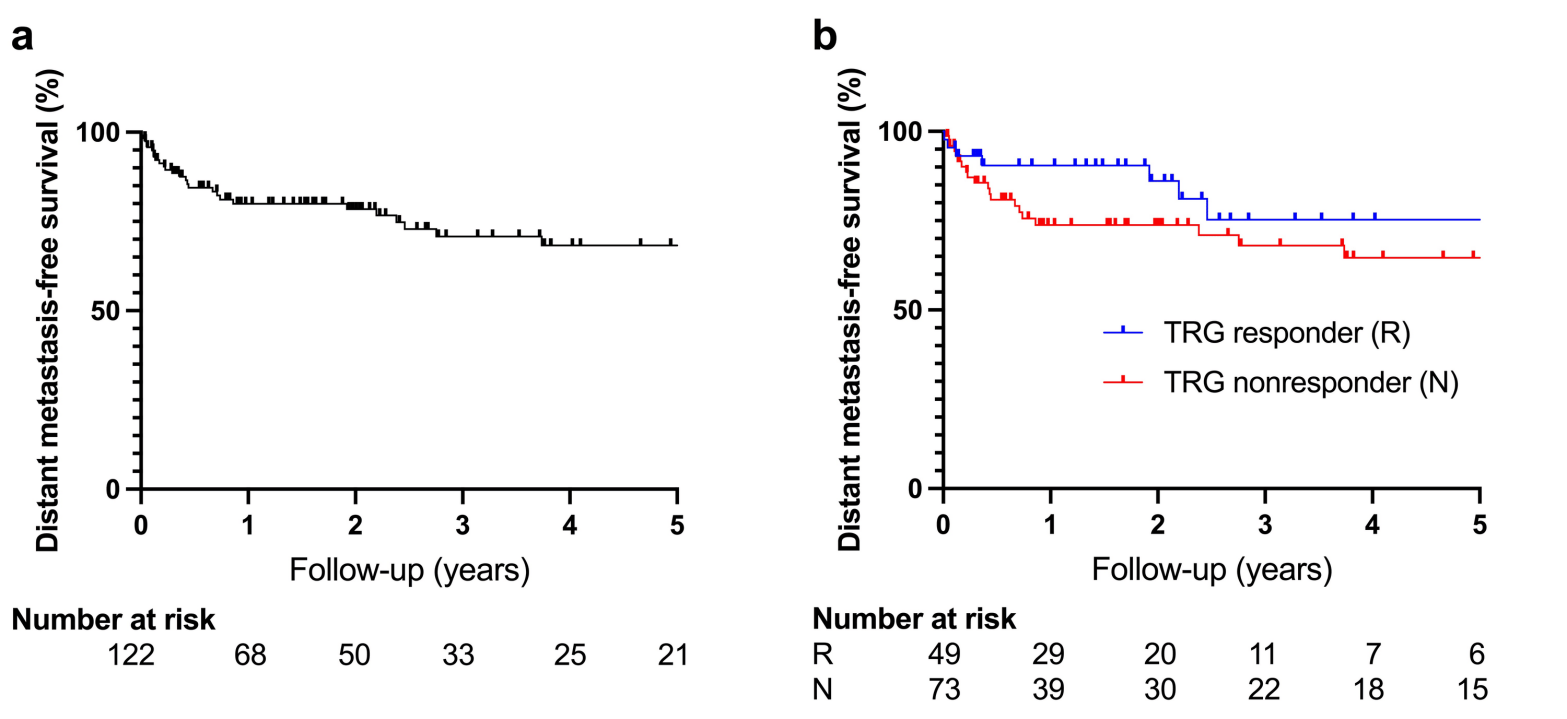
Distant metastasis-free survival (a) Distant metastasis-free survival for the whole study cohort. (b) Distant metastasis-free survival according to TRG response. TRG: tumor regression grading

The median clinical follow-up was 21.4 months (IQR 7.5 to 46.5; range 0.5 to 118.1), and the median radiographic follow-up was 18.7 months (IQR 4.3 to 44.7; range 0.6 to 118.1). Overall, LC was high, with a one-year LC of 94.8% (95% confidence interval (CI) 88.1 to 97.8). Three-year and five-year LC were 92.7% (95% CI 83.7 to 96.8) and 92.7% (95% CI 83.7 to 96.8), respectively (Figure 2A). Comparatively, the one-year PFS was lower at 76.1% (95% CI 66.6 to 83.2), with three-year and five-year PFS rates of 65.3% (95% CI 53.4 to 74.8) and 62.9% (95% CI 50.4 to 73.0), respectively (Figure 3A). Similarly, DMFS was 79.9% (95% CI 70.8 to 86.5) at one year, 70.8% (95% CI 59.1 to 79.7) after three years, and 68.3% (95% CI 55.7 to 77.8) after five years (Figure 4A). The median OS was not reached. The one-year, three-year, and five-year OS were 96.5% (95% CI 91.1 to 98.7), 92.8% (95% CI 83.7 to 96.9), and 92.8% (95% CI 83.7 to 96.9), respectively. During the available follow-up, nine local failures were detected (7.4%), 26 patients developed distant metastases (21.3%), and six patients died (4.9%). Newly diagnosed metastases were mostly located in the lungs (61.5%), followed by the liver (26.9%), the brain (7.7%), and distant lymph nodes (3.8%). Distant metastases were the main drivers for PFS and DMFS outcomes. Multivariable Cox regression revealed a poorer PFS for TRG nonresponders than for responders (hazard ratio (HR): 2.55; p=0.028) (Figure 3B). A comparable finding regarding TRG response was observed for LC (HR: 4.79; p=0.065), albeit not significant (Figure 2B). The TRG nonresponders had a higher rate of distant metastasis, though not formally statistically significant (HR: 2.18; p=0.073) (Figure 4B). Moreover, the localization in the lower third of the rectum was associated with lower LC (HR: 5.70; p=0.044). Additionally, patients with positive lateral lymph nodes had a higher rate of progression (HR: 2.37; p=0.081) (Figure 3C) and distant metastases (HR: 2.35; p=0.123), although not statistically significant. Multivariable Cox regression analyses did not identify other significantly associated factors for LC, PFS, and DMFS. Respective log-log plots and testing based on Schoenfeld residuals indicated that the proportional hazards assumption was not violated across all Cox regression models. Outcomes are summarized in Table 2. Results of the Cox regression are summarized in Table 3 for PFS and DMFS and in Table 4 for LC.

**Table 2:** Oncological outcomes DMFS: distant metastasis-free survival, IQR: interquartile range, LC: local control, OS: overall survival, PFS: progression-free survival

Parameters	N	%
Follow-up (months)		
Median clinical follow-up (IQR; range)	21.4 (7.5-46.5; range 0.5-118.1)	
Mean clinical follow-up	31	
Median radiographic follow-up (IQR; range)	18.7 (4.3-44.7; range 0.6-118.1)	
Mean radiographic follow-up	27.7	
Survival		
5-year LC		92.7
5-year PFS		62.9
5-year DMFS		68.3
5-year OS		92.8
Reported death cases	6	4.9
Due to postoperative complications	3	2.5
Due to other causes	2	1.6
Due to disease progression	1	0.8
Local recurrence	9	7.4
Distant metastases	26	21.3
Lungs	16	13.1
Liver	7	5.7
Brain	2	1.6
Distant lymph nodes	1	0.8

**Table 3:** Multivariable Cox regression for progression-free survival and distant metastasis-free survival CRM: circumferential resection margin; EMVI: extramural vascular invasion; HR: hazard ratio; CI: confidence interval; lat. LN: lateral lymph node; N/A: not applicable; TRG: tumor regression grading; (-): negative; (+): positive

Variables	Progression-free survival			Distant metastasis-free survival		
	HR	95% CI	p	HR	95% CI	p
Tumor stage						
cT2, cT3	reference			reference		
cT4	0.73	(0.26 to 1.85)	0.52	0.43	(0.11 to 1.29)	0.16
General node stage						
cN0	reference			reference		
cN+	0.72	(0.31 to 1.75)	0.45	0.71	(0.30 to 1.83)	0.46
cN2 stage						
cN0, cN1	reference			reference		
cN2	0.51	(0.16 to 1.40)	0.22	0.51	(0.13 to 1.52)	0.26
Lat. LN stage						
(-) lat. LN	reference			reference		
(+) lat. LN	2.37	(0.86 to 6.19)	0.08	2.35	(0.75 to 6.82)	0.12
Localization						
Other	reference			N/A		
Lower rectum	1.13	(0.55 to 2.32)	0.73	N/A		
CRM						
(-) CRM	reference			reference		
(+) CRM	1.15	(0.43 to 3.30)	0.77	1.37	(0.44 to 4.80)	0.59
EMVI						
(-) EMVI	reference			reference		
(+) EMVI	0.5	(0.07 to 1.84)	0.36	0.62	(0.09 to 2.34)	0.54
Risk groups						
Low risk	reference			reference		
High risk	1.38	(0.41 to 4.31)	0.58	1.17	(0.29 to 4.26)	0.81
TRG response						
Responder	reference			reference		
Nonresponder	2.55	(1.16 to 6.42)	0.02	2.18	(0.97 to 5.55)	0.07
Age (years)	1	(0.97 to 1.03)	0.93	1	(0.97 to 1.03)	0.69

**Table 4:** Multivariable Cox regression for local control CRM: circumferential resection margin; HR: hazard ratio; CI: confidence interval; lat. LN: lateral lymph node; TRG: tumor regression grading; (-): negative; (+): positive

Variables	Local control		
	HR	95% CI	p
Tumor stage			
cT2, cT3	reference		
cT4	1.44	(0.23 to 9.31)	0.68
General node stage			
cN0	reference		
cN+	2.06	(0.24 to 44.38)	0.54
cN2 stage			
cN0, cN1	reference		
cN2	0.27	(0.01 to 1.97)	0.26
Lat. LN stage			
(-) lat. LN	reference		
(+) lat. LN	2.1	(0.31 to 13.5)	0.41
Localization			
Other	reference		
Lower rectum	5.7	(1.19 to 42.2)	0.04
CRM			
(-) CRM	reference		
(+) CRM	1.02	(0.15 to 8.43)	0.97
Risk groups			
Low risk	reference		
High risk	2.38	(0.15 to 34.14)	0.51
TRG response			
Responder	reference		
Nonresponder	4.79	(0.90 to 25.36)	0.06

Adverse events

Adverse events according to the Common Terminology Criteria for Adverse Events v5.0 are summarized in Table 5 [15]. Most patients tolerated the radiochemotherapy well. Six patients discontinued the radiotherapy because of acute adverse events (4.9%). Out of the patients receiving consolidative chemotherapy, 12 patients required chemotherapy dose reduction (38.7%). The most common acute adverse events related to neoadjuvant therapy were fatigue (46.7%) and pain (39.3%). Twenty-two patients developed peripheral neuropathy (18%). The most common hematological adverse effect was anemia (22.1%). Additionally, three cases of febrile neutropenia were reported (2.5%). Elevated liver enzymes were reported in 26.2%. Acute kidney injury affected six patients (4.9%). Forty-three patients had postoperative complications requiring an invasive intervention, according to Clavien-Dindo III (35.2%). Five patients received a permanent colostomy (4.1%). Furthermore, three patients died because of postoperative complications (2.5%). Comparatively, the most reported long-term adverse event during the follow-up period was peripheral neuropathy (14 patients, 11%).

**Table 5:** Adverse events

Variable	N	%
General acute adverse events		
Fatigue	57	46.7
Pain	48	39.3
Nausea	32	26.2
Diarrhea	27	22.1
Obstipation	17	13.9
Dyspnea	13	10.7
Rectal hemorrhage	12	9.8
Hematological acute adverse events		
Anemia	27	22.1
Leukocytopenia	17	13.9
Neutropenia	6	4.9
Febrile neutropenia	3	2.5
Thrombocytopenia	4	3.3
Infectious adverse events		
Infection	22	18
Anorectal infection	13	10.7
Dermatitis radiation	13	10.7
Rectal mucositis	8	6.6
Cystitis	3	2.5
Other acute adverse events		
Elevated liver enzymes	32	26.2
Peripheral sensory neuropathy	22	18
Fecal incontinence	8	6.6
Rectal fistula	7	5.7
Acute kidney injury	6	4.9
Urinary incontinence	5	4.1
Hepatic failure	4	3.3
Rectal stenosis	4	3.3
Erectile dysfunction	3	2.5
Postoperative acute adverse events		
Severe complications requiring intervention (Clavien-Dindo IIIa/b)	43	35.2
Clavien-Dindo IIIb	27	22.1
Wound dehiscence	35	28.7
Anastomosis insufficiency	21	17.2
Permanent colostomy	5	4.1
Clavien-Dindo V	3	2.5
Chronic adverse events during follow-up		
Peripheral sensory neuropathy	14	11
Erectile dysfunction	1	0.8
Anorectal infection	1	0.8
Fecal incontinence	1	0.8

## Discussion

We observed high patient treatment adherence across TNT and LC-RCT in a predominantly high-risk cohort. Regarding the evaluation of our retrospective results, a wide array of clinical trials have confirmed a PFS and DMFS benefit for TNT compared to protocols utilizing preoperative concomitant radiochemotherapy and adjuvant chemotherapy [3,5,16,17]. Based on these studies, the RAPIDO and Partenariat de Recherche en Oncologie Digestive (PRODIGE) 23 protocols have become well-established in clinical practice [3,5]. The RAPIDO protocol utilizes a SC-TNT protocol (5 x 5 Gy) with consolidative FOLFOX or CAPOX, and the PRODIGE 23 protocol is an LC-TNT protocol with inductive FOLFIRINOX followed by LC-RCT [3,5]. Both trials demonstrated benefits in disease-related treatment failure (DRTF) and disease-free survival (DFS) for TNT. However, there were significant differences in patient characteristics, such as the presence of a threatened or involved CRM (61.6% vs. 25.9% for RAPIDO and PRODIGE 23, respectively) [3,5].

Concerning survival outcomes, the RAPIDO trial reported a three-year DRTF of 23.7% for the TNT arm and 30.4% for the conventional arm utilizing neoadjuvant long-course radiochemotherapy and adjuvant consolidation [3]. Follow-up data reported a slightly lower five-year DRTF of 27.8% and a distant metastasis rate of 23.0% for the TNT arm [18,19]. Another prospective cohort trial, Locally Advanced Rectal Cancer Treatment - Uppsala Style (LARCT-US), based on the RAPIDO protocol, reported similar results for DRTF (28%) [20]. Similarly, the PRODIGE 23 trial reported a three-year DFS of 75.7% for TNT and 68.5% for the control arm [5]. Our retrospective data revealed lower but overall comparable PFS outcomes, with three-year PFS at 65.3% and five-year PFS at 62.9%, nonetheless staying within the range of the prospective studies.

RAPIDO and PRODIGE 23 reported a three-year local recurrence of 8.3% and 4.3% for the TNT arm [3,5]. Our results demonstrate similar rates of local recurrence (Table 2). Notably, the five-year follow-up of the RAPIDO trial reported rising local recurrence rates in the TNT arm (10.2% vs. 6.1%) [18,19]. The cohort receiving TNT was found to be associated with worse surgical quality (i.e., breach of mesorectum during surgery, 11.1% and 6.4% for the TNT and control arm, respectively) [19]. A similar drop in LC was not observed in our study, although a similar rate of 9% regarding incomplete mesorectum resection (i.e., Mercury grading 2 and 3) was observed. Regarding distant metastases, both prospective studies reported similar rates at three years (20.0% in RAPIDO vs. 16.9% in PRODIGE 23) for the experimental arm [3,5]. The PRODIGE 23 trial reported a three-year DMFS of 78.8% for the TNT arm and 71.7% for the control arm [5]. Comparatively, the distant metastasis rate of our cohort was around 21.3%, with a three-year DMFS of 70.8%. Regarding OS, the RAPIDO trial reported a five-year OS of 81.7% for the experimental arm and 80.2% for the control arm, respectively [18,19]. Furthermore, the PRODIGE 23 trial reported the three-year OS to be 90.8% and 87.7%, without significant survival benefits for either group [5]. Still, an OS benefit for the TNT arm was reported within the seven-year follow-up of the PRODIGE 23 trial (81.9% vs. 76.1% for the TNT and standard cohort, respectively) [21]. The third study, LARCT-US, reported an 88% OS rate [20]. In comparison, the five-year OS in our cohort was 92.8%. Overall, similar results are also reported in the analysis of the CAO/ARO/AIO-12 trial, which demonstrated a lack of long-term OS benefit for the TNT group [22,23].

Conclusively, the outcomes of our retrospective cohort study mostly remain consistent with the prospective results of the RAPIDO and PRODIGE 23 trials (Tables 6-7). Outcome differences, particularly for PFS and DMFS, may be attributed to the aforementioned variations in patient characteristics and high-risk markers. As mentioned above, RAPIDO reported high rates of CRM positivity (61.6% vs. 60.2% for the TNT and control arm, respectively) and PRODIGE 23 described lower rates for CRM (25.9 vs. 27.7% for the TNT and control arm, respectively) [3,5]. Comparatively, our study reported a CRM positivity rate of 36.9%. Furthermore, EMVI positivity was 12.3% in our cohort, whereas RAPIDO reported significantly higher rates (32.0% vs. 27.8%) [3]. PRODIGE 23 did not report any EMVI data at all [5]. Additionally, both prospective studies included patients receiving adjuvant chemotherapy in the follow-up period (RAPIDO 1.4% vs. 46.9%, PRODIGE 23 77.3% vs. 78.6%) [3,5]. Patients in our cohort did not receive any adjuvant therapy, as defined in the exclusion criteria. Similarly, it might be hypothesized that prospective trial populations, which are often subject to strict selection criteria and treatment protocols, cannot be entirely generalized to an all-comers population of patients. TNT was established within ESMO guidelines around 2020 [6]. As such, a subgroup of high-risk cases did receive long-course radiochemotherapy without adjuvant chemotherapy, in accordance with previous ESMO guidelines [5]. Furthermore, the discontinuation of consolidative chemotherapy in our cohort (12 patients, 38.7%) could have affected outcomes. For instance, the post hoc analysis of the CAO/ARO/AIO-04 trial found that higher adherence to neoadjuvant chemotherapy was associated with a better DFS outcome [24]. Regarding the treatment adherence to adjuvant chemotherapy, this was not the case [24]. Finally, our median follow-up was 21.4 months, which is shorter than the follow-up in both prospective trials. Commonly, patients prefer to undergo cancer treatment at academic cancer centers and switch to local care providers after the initial follow-up. As such, our follow-up is limited and must be considered when interpreting our results. These factors could be possible causes for the PFS and DMFS differences between our cohort and the prospective results.

**Table 6:** Comparison of three-year outcomes *First percentage and second percentage are for the TNT and control arm, respectively (i.e., TNT vs. control). DFS: disease-free survival; DM: distant metastasis; DMFS: distant metastasis-free survival; DRTF: disease-related treatment failure; LR: locoregional recurrence; OS: overall survival; PFS: progression-free survival; TNT: total neoadjuvant therapy; PRODIGE: Partenariat de Recherche en Oncologie Digestive; RAPIDO: Rectal Cancer and Preoperative Induction Therapy Followed by Dedicated Operation

Survival parameters	Bahadoer et al. 2021 (RAPIDO) [3]	Conroy et al. 2021 (PRODIGE 23) [5]	This study
Local recurrence	LR 8.3% vs. 6.0%*	LR 4.3% vs. 5.6%	LR 7.3%
Progression	DRTF 23.7% vs. 30.4%*	DFS 75.7% vs. 68.5%	PFS 65.3%
Distant metastasis	DM 20.0% vs. 26.8%*	DMFS 78.8% vs. 71.7%	DMFS 70.8%
Overall survival	OS 89.1% vs. 88.8%*	OS 90.8% vs. 87.7%	OS 92.8%

**Table 7:** Comparison of five-year outcomes *First percentage and second percentage are for the TNT and control arm, respectively (i.e., TNT vs. control). DM: distant metastasis; DMFS: distant metastasis-free survival; DRTF: disease-related treatment failure; LR: locoregional recurrence; N/A: not applicable; OS: overall survival; PFS: progression-free survival; TNT: total neoadjuvant therapy; PRODIGE: Partenariat de Recherche en Oncologie Digestive; RAPIDO: Rectal Cancer and Preoperative Induction Therapy Followed by Dedicated Operation

Survival parameters	Bahadoer et al. 2023, Dijkstra et al. 2023 (RAPIDO) [18,19]	Conroy et al. 2021 (PRODIGE 23) [5]	This study
Local recurrence	LR 10.2% vs. 6.1%*	N/A	LR 7.3%
Progression	DRTF 27.8% vs. 34.0%*	N/A	PFS 62.9%
Distant metastasis	DM 23.0% vs. 30.4%*	N/A	DMFS 68.3%
Overall survival	OS 81.7% vs. 80.2%*	N/A	OS 92.8%

Concerning pCR and the potential for organ preservation, RAPIDO and PRODIGE 23 reported comparable pCR data for the TNT arm (28% vs. 28% for the RAPIDO and PRODIGE 23 trials, respectively) [3,5]. In comparison, the pCR (i.e., Dworak TRG 4) rate in this retrospective study was 28.7%. Furthermore, all patients within our cohort underwent surgery. As such, a statement regarding the potential of organ preservation (i.e., which patient would have potentially benefited) is not feasible.

However, the most effective and suitable TNT regimen, as well as reliable indication guidelines, remain an ongoing subject of discussions and clinical trials. Several clinical guidelines have introduced indications for the use of TNT. Most commonly utilized as an indication for TNT is the high-risk constellation defined within the RAPIDO trial (CRM and/or EMVI positivity, T4 stage, N2 or higher lymph node stage, or positive lateral lymph nodes) [3,25]. The ESMO guideline established the risk classifications "intermediate," "advanced," and "bad" as indications for TNT, which are similar to the RAPIDO criteria [6,25]. Likewise, the NCCN established a broad TNT indication for all LARC cN+ settings, citing survival benefits across all risk classifications [7]. In addition, both guidelines and various systematic reviews do not give distinct recommendations for a particular radiation concept or a chemotherapy protocol [2,6,7,25,26]. For instance, the PRODIGE 23 trial was criticized for using FOLFIRINOX instead of FOLFOX, as the survival benefit of adding irinotecan was not clearly proven [2,5,25,26]. Regarding the variation of TNT protocols, the Organ Preservation for Rectal Adenocarcinoma (OPRA) study compared LC-TNT with either induction or consolidation chemotherapy, reporting little to no differences in DFS, DMFS, and OS [27]. Therefore, the differentiation in indication for SC-TNT and LC-TNT remains unclear and subject to institutional preferences and tumor boards. A comparable phase III study (ACO/ARO/AIO-18.1) comparing SC-TNT to LC-TNT is currently ongoing (NCT04246684; registration date: January 28, 2020) [28].

Consequently, no clear markers to differentiate TNT and conventional protocols were identified in the prospective clinical trials or current systematic reviews [2,25,26]. Similarly, the outcome of our retrospective cohort presents a similar lack of valid prognostic clinical markers, except for the already known positive lateral lymph nodes for PFS and lower rectum localization for LC. A known limitation of previous phase III studies comparing TNT and conventional radiochemotherapy protocols was the lack of comparability in patient characteristics and the use of high-risk classifications, as described above [25,26]. The relatively broad TNT indications of the guidelines mentioned above, combined with the relative lack of clear prognostic factors, highlight the potential risk of over- and undertreatment [25]. Significant differences regarding toxicity exist for TNT and other radiation protocols. Compared to concomitant radiochemotherapy, neoadjuvant FOLFOX causes higher rates of toxicity, such as hematological side effects and peripheral polyneuropathy [2,3,5,25]. Therefore, further research is warranted to determine the optimal patient selection for both treatment strategies, ultimately identifying the most reliable prognostic factors indicating TNT.

Herein, we found differences in PFS between TRG responders and nonresponders. TRG grouping could, therefore, represent a potential factor in determining the use of postoperative chemotherapy to decrease the risk of early disease progression. As previous studies, such as the aforementioned PRODIGE 23 trial, had a high number of patients receiving adjuvant chemotherapy within the TNT and conventional cohorts, the significance of the already established gradings, such as TRG, might have been underestimated [5].

Nevertheless, valid predictive factors to guide treatment decisions between TNT and conventional radiochemotherapy are the focus of ongoing research. Besides conventional clinical factors, molecular and immunological factors seem promising in refining TNT algorithms. For example, the pathological upregulation of nuclear factor erythroid 2-related factor 2, a regulatory protein that controls the expression of antioxidant mechanisms, was identified as a protective factor against radiation in malignant cells [29]. Furthermore, the tumor microenvironment, its composition, and tumor-cell interaction have a significant impact on therapy resistance [30]. For instance, the interleukin-1 pathway was identified as a mechanism that could drive radiation resistance through fibroblast activation [30]. Correspondingly, organoid models for rectal cancer are currently being investigated for in vitro chemotherapy and radiation sensitivity testing [30]. In future research, these prospective factors will need to be validated in clinical settings. Currently, TNT is being tested in combination with immunotherapy and targeted therapies, such as anti-VEGF antibodies [31]. LARC cases with mismatch repair deficiency are already successfully treated with dostarlimab monotherapy, a programmed cell death protein 1 (PD-1) inhibitor [6]. Research combining TNT and PD-1 inhibitors for common microsatellite-stable LARC cases is ongoing [31]. As neoadjuvant radiochemotherapy is a safe and tolerable treatment for most patients, the identification of such novel markers is necessary for future clinical decision-making.

This study has certain limitations pertinent to its design. First, it is a retrospective study, leading to inherent biases and residual confounding. Moreover, some of the patient, tumor, and treatment characteristics were not available for all patients. This issue also applies to the toxicity grading, as it was not comprehensively reported for many common low-grade adverse events, such as nausea or fatigue. Additionally, the short overall follow-up period needs to be acknowledged. Finally, the small number of events lowers the statistical power of the regression analyses, preventing the detection of subtle associations between the co-variables and outcomes. All limitations have to be considered when interpreting the results of this work.

## Conclusions

Neoadjuvant radiochemotherapy, either as TNT or conventional LC-RCT, appears to be a safe, tolerable, and effective treatment option for LARC. The TRG response could potentially be used as a variable for further risk stratification. As study results and guidelines have established the indication of TNT for LARC, future research is needed to identify reliable predictive and prognostic factors to ensure adequate patient selection, ultimately avoiding over- and undertreatment and ensuring organ preservation.
